# Genomic insights into the antibiotic resistance pattern of the tetracycline-degrading bacterium, *Arthrobacter nicotianae* OTC-16

**DOI:** 10.1038/s41598-021-94840-y

**Published:** 2021-08-02

**Authors:** Xin Zhang, Rongrong Zhu, Weilin Li, Junwei Ma, Hui Lin

**Affiliations:** 1 College of Forest and Biotechnology, Zhejiang A & F University, Hangzhou, 311300 China; 2 The Institute of Environment, Resources, Soil and Fertilizers, Zhejiang Academy of Agricultural Sciences, Hangzhou, 310021 China

**Keywords:** Environmental impact, Environmental microbiology, Gene expression, Microbiology, Natural hazards

## Abstract

Although many bacteria have the potential to remove antibiotic residues from environmental niches, the benefits of using antibiotic-degrading bacteria to manage antibiotic pollution should be assessed against the risk of the potential expansion of antimicrobial resistance. This study investigated the antibiotic resistance pattern of the bacterium *Arthrobacter nicotianae* OTC-16, which shows substantial biodegradation of oxytetracycline (OTC)/tetracycline. The results showed that this strain could be resistant to at least seven categories of 15 antibiotics, based on antimicrobial susceptibility testing. The genome of *A. nicotianae* OTC-16 contains one chromosome (3,643,989 bp) and two plasmids (plasmid1, 123,894 bp and plasmid2, 29,841 bp). Of the 3,561 genes isolated, eight were related to antibiotic resistance. During OTC degradation by the strain OTC-16, the expression of *ant2ia*, *sul1*, *tet33*, and *cml_e8* in the plasmid, and one gene (*tetV*) in the chromosome were tracked using real-time quantitative reverse transcription-polymerase chain reaction (qRT-PCR). Only the plasmid-derived resistance genes were up-regulated in the presence of OTC. The presence of OTC increased the tolerance of strain OTC-16 to streptomycin sulphate. The findings of this study can help deepen our understanding of the behavioural characteristics of resistance genes and adaptive evolution of drug-resistant bacteria.

## Introduction

Antibiotics have received global attention owing to their wide application in human therapy and livestock agriculture and their wide occurrence in terrestrial and aquatic environments^1,2^. However, this has led to a measurable increase in antibiotic resistance genes (ARGs) and the potential spread to clinics. Environmental bacteria can utilise antibiotics as carbon or nitrogen sources. For example, there are specific antibiotic-degrading bacteria for β-lactams^3^, tetracyclines^4^, and sulphonamides^5^. These antibiotic-degrading bacteria have the potential to be developed for bioaugmentation/bioremediation of antibiotic-contaminated environments^3,4^ such as soils near pharmaceutical manufacturers, pharmaceutical wastewater, or livestock manure^6^. However, most bioaugmentation/bioremediation agents are applied to facilitate the removal of antibiotics at fixed-point sites, such as wastewater plants. One important reason for limiting the widespread use of biodegrading agents is that the strains are ecologically risky. Strains with the capability to degrade antibiotics are usually antibiotic-resistant and might carry some mobile ARGs. Numerous reports have demonstrated that naked DNA carrying ARGs may exist for a long time even after the death of the bioaugmentation agents, thus endangering human health through various pathways such as direct contact or pollution of the food chain^7^. In addition, the horizontal exchange of ARGs through mobile genetic elements such as integrons, plasmids, and transposons would increase resistance rapidly in many indigenous environmental bacteria owing to exposure to resistant strains^8,9^. Because of these high ecological risks, the World Health Organization has listed ARGs as one of the most important challenges threatening human health in the twenty-first century. Therefore, characterisation of the antibiotic resistance profile of these microbial inoculants is an essential step for assessing the ecological risks before their application to guarantee their safe use. Current researches on antibiotic-degrading strains are mostly limited to the degradation characteristics, metabolic pathways, and degradation-based genomic analysis of the strains^10,11^. However, very few studies on the antibiotic-resistant pattern and intrinsic mechanism of these antibiotic-degrading strains have been reported, which are extremely important in assessing their ecological risk after environmental release.

We previously isolated tetracycline antibiotic-degrading bacterium *Arthrobacter nicotianae* OTC-16 from activated sludge. The strain shows interesting biodegradation of oxytetracycline (OTC)/tetracycline in various matrices, such as in aqueous media under a wide range of pH, temperature, and antibiotic concentration; manure samples from different animals; and soils with different properties^12^. Preliminary analysis^12^ showed that the application of strain OTC-16 did not increase some commonly studied tetracycline-resistant genes in the soil, such as *tetW*, *tetB*, *tetA,* and *tetG*, and could effectively inhibit the proliferation of *tetW* and *tetB*. Nonetheless, these results are insufficient to conclude that there is no risk in applying this degrading bacterium in the environment. The aim of the study was to investigate the antibiotic resistance of the antibiotic-degrading strain OTC-16 from a genomics perspective, thereby offering an alternative idea for further genetic engineering modification of the strain to decrease its ecological risk. The results will deepen our understanding of the resistance pattern of antibiotic-degrading strains and elucidate the role of ARGs in antibiotic resistance and adaptive evolution.

## Results and discussion

### Antimicrobial susceptibility analysis

*A. nicotianae* OTC-16 was previously isolated for its efficient biodegradation of OTC and tetracycline, indicating that the strain was resistant to these two antibiotics. Antimicrobial susceptibility testing further confirmed that strain OTC-16 also exhibited obvious resistance to 13 other tested antibiotics, namely, sulfamethoxine, sulfamethoxazole, sulfamethazine, sulfamonomethoxine, cotrimoxazole, bacitracin, streptomycin, streptomycin sulphate, nalidixic acid, nystatin, furazolidone, isopropyl-b-D-thiogalactoside, and metronidazole. In addition, strain OTC-16 showed low resistance to vancomycin, macrodantin, polymyxin, and kanamycin (Table 1). Overall, strain OTC-16 showed multiple resistance to antimicrobial agents. *Arthrobacter* strains have been reported to show resistance to various pharmaceuticals and their metabolites, such as sulfadiazine^13^, kanamycin^14^, and ritalinic acid^15^. In addition, *Arthrobacter* species are widely used in the bioremediation of other pollutants, such as atrazine and 1-naphthol^16–18^. It is assumed that *Arthrobacter* strains have powerful enzyme systems for decomposing heterogeneous compounds. Aside from the multiple degradation property, their high adaptation capacity and rapid response to environmental changes^14^ confer greater application potential to these strains in the natural attenuation of various classes of pollutants in the environment.

**Table 1:** Inhibitory effect of tested drugs on growth of strain OTC-16.

Antibiotic categories	Antibiotics (μg per disk)	Test effect	Antibiotic categories	Antibiotics (μg per disk)	Test effect
Tetracyclines	Chlorotetracycline(30)	+	Glycopeptides	Vancomycin(30)	−
	Doxycycline(30)	−		Polymyxin(300)	+
	Oxytetracycline(30)	++	Polypeptide	Bacitracin(0.04)	++
	Tetracycline(30)	++		Vancomycin(30)	+
Sulfonamides	Sulfadimethoxine(100)	++	Lincosamides	Clindamycin(2)	−
	Sulfamethoxazole(30)	++		Lincomycin(20)	+
	Sulfadimethazine(300)	++	Nitrofurans	Macrodantin(300)	+
	Sulfamonomethoxine(30)	++		Furazolidone(300)	++
	Cotrimoxazole(23.75)	++	Polyene	Nystatin(100)	++
Macrolides	Erythromycin(15)	−	Quinolones	Ciprofloxacin(5)	−
	Midecamycin(30)	−		Ciprofloracin(5)	−
Aminoglycosides	Gentamicin(10)	−		Ofloxacin(5)	−
	Amikacin(30)	−		Enoxacin(10)	−
	Tobramycin(10)	+		Enrofloxacin(10)	−
	Neomycin(30)	−		Norfloxacin(10)	−
	Kanamycin(30)	+		Nalidixic acid(30)	++
	Kanamycin sulphate(30)	+	Penicillins	Piperacillin(100)	−
	Streptomycin(10)	++		Ampicillin(20)	−
	Streptomycin sulphate(10)	++		Amoxicillin(20)	−
Cephalosporin	Cefotaxime(30)	−		Penicillin(20)	−
	Aventiamycin IV(30)	−		Oxacillin(20)	−
	Cefalexin(30)	−	Chloramphenicol	Chloramphenicol(30)	−
	Ceftriaxone(30)	−	Others	Rifampicin(5)	−
	Mefoxin(30)	−		Metronidazole(5)	++
	Cefoperazone(75)	−		Isopropyl-b-D-thiogalactoside(0.01 g/ml)	++

Note: -: Strain OTC-16 is highly sensitive to the drugs (diameter of bacteriostatic circle ≥ 1.3 cm); ++: strain OTC-16 is high resistant to the drugs (No bacteriostatic circles were observed); +: strain OTC-16 is medium resistant to the drugs (diameter of bacteriostatic circle < 1.3 cm).

### Genomic features of *A. nicotianae* OTC-16

**Figure 1:**
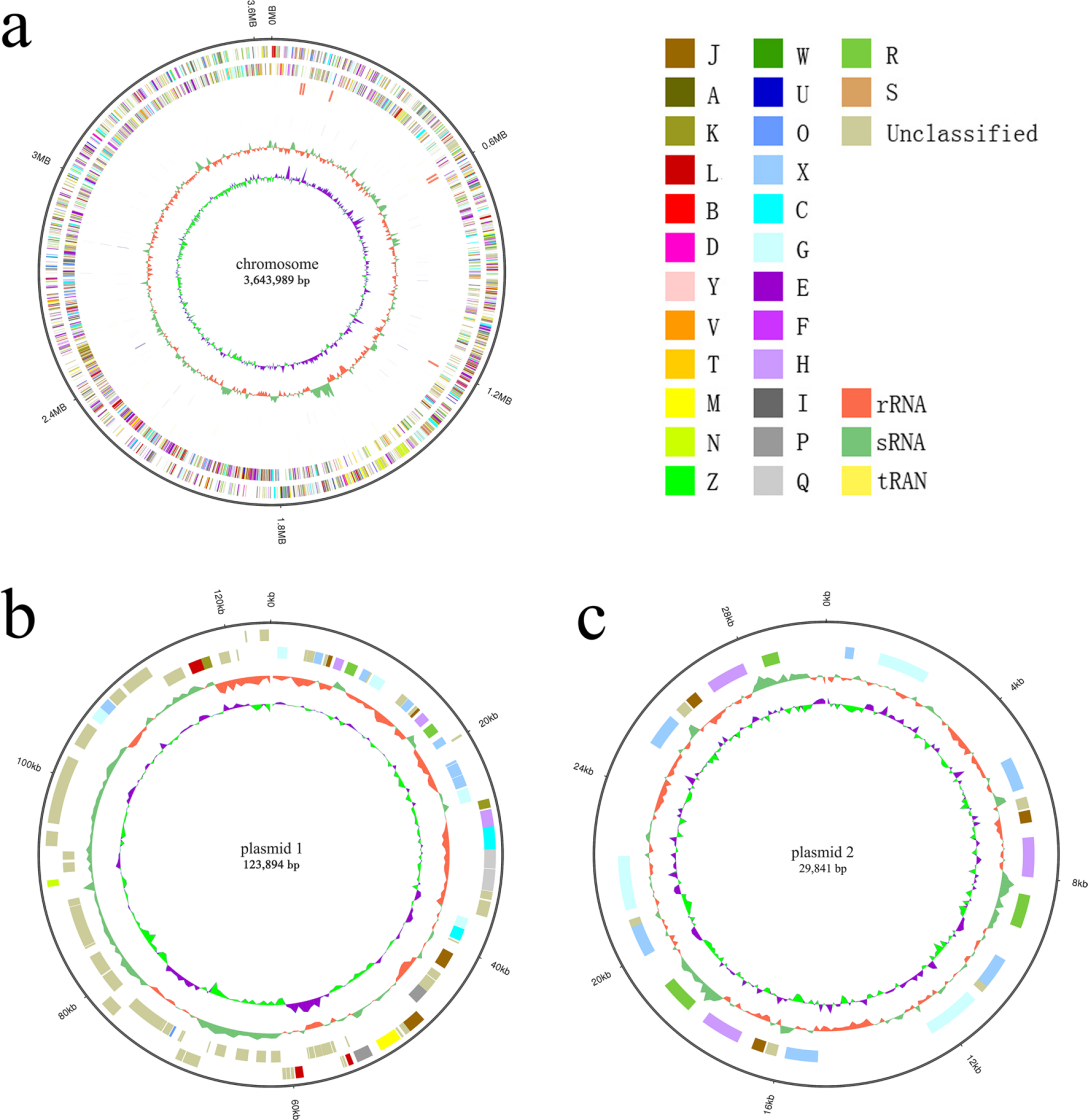
Circular map of the chromosome (**a**), plasmid1 (**b**) and plasmid2 (**c**) of strain OTC-16. From inner to outer: Genome size; Forward Strand Gene(coloured by COG classification); Reverse Strand Gene(coloured by COG classification); Forward Strand ncRNA; Reverse Strand ncRNA; Repeat gene; G + C content; GC-SKEW(calculated as (G-C)/(G + C); green/purple peaks out/inside the circle indicate values higher or lower than 1, respectively.

**Figure 2:**
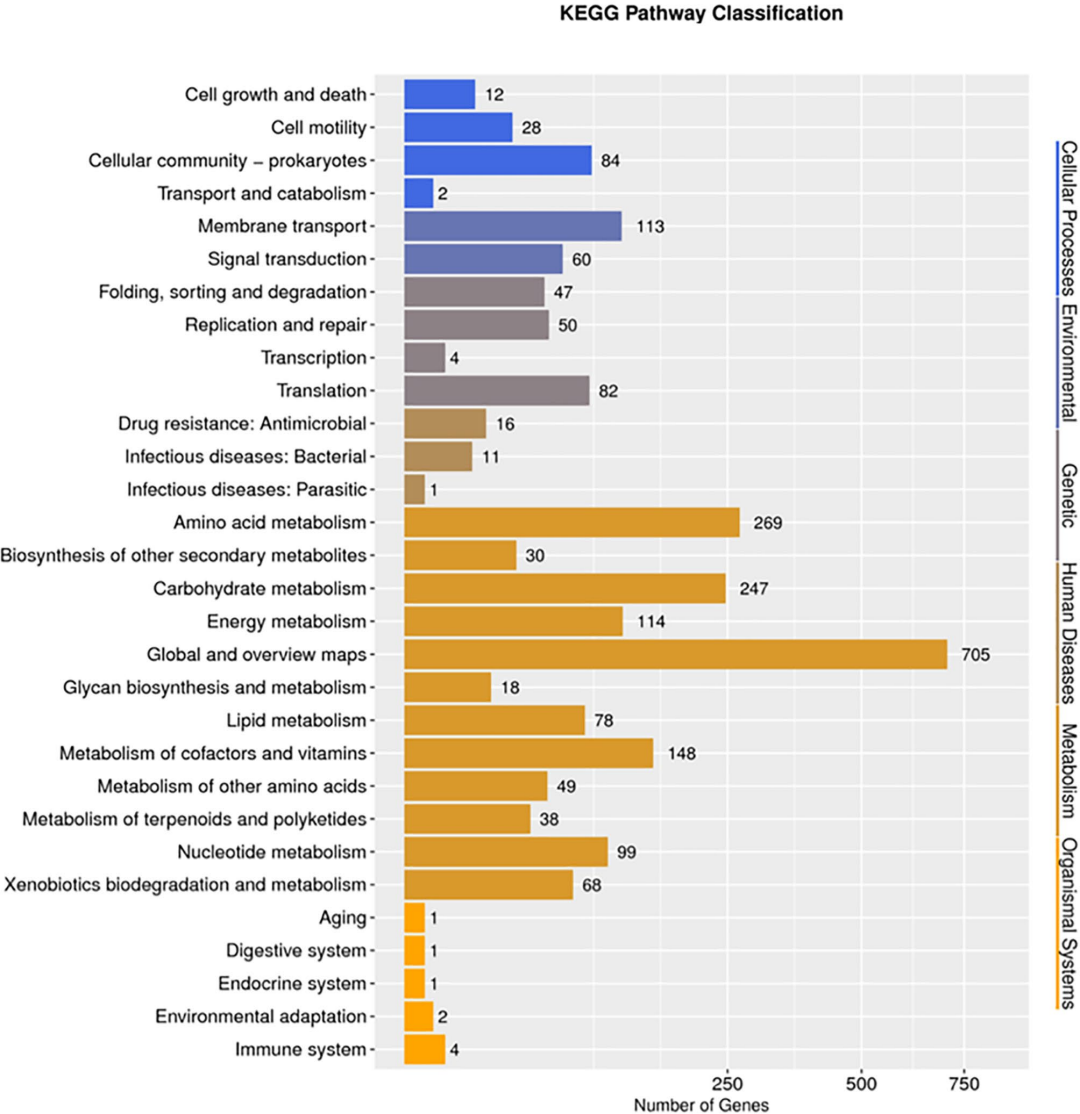
KEGG function annotation distribution map of strain OTC-16.

The whole genome of the newly isolated *A. nicotianae* OTC-16 was sequenced (accession number NZ_CP 033 081). Briefly, *A. nicotianae* OTC-16 contained one chromosome with a total length of 3,643,989 bp and two plasmids with lengths of 123,894 bp and 29,841 bp, respectively (Fig. 1); the GC-content of the genome was 61.71%. This genome contained 3,561 genes, including 67 tRNA, 19 rRNA, and 28 sRNA, with a total length of 379,7724 bp. Figure S1 shows the detailed Gene Ontology (GO) functional information from which three main functional domains were established: (1) cellular component, (2) molecular function, and (3) biological process. A total of 3429 genes were found to be enriched in biological processes; 1326 genes were involved in metabolic processes, 992 genes were involved in cellular processes, and 132 genes responded to stimuli. The statistics of Kyoto Encyclopedia of Genes and Genomes (KEGG) annotation showed that the genes of strain OTC-16 can be classified into six categories, with 30 genes related to the biosynthesis of secondary metabolites, 12 genes involved in infectious diseases, 68 genes involved in xenobiotics biodegradation and metabolism, and 16 genes related to drug resistance (Fig. 2). These drug resistance genes may contribute to the wide resistance of strain OTC-16 to antimicrobials.

The ARGs were obtained using Comprehensive Antibiotic Research Database (CARD) analysis, and detailed information is listed in Table S1. Briefly, eight antibiotic resistance types, a total of 23 copies of ARGs of strain OTC-16 were recognised, with most of them, four types (*sul1*, *tet33*, *ant2ia*, and *cml_e8*) and 15 copies of ARGs, located in plasmids. The cells of strain OTC-16 grew well when exposed to all the possible antibiotics encoded by the ARGs, as was observed in antimicrobial susceptibility testing. Tetracycline resistance involves *tet33* in both plasmids 1 and 2 and *tetV* in the chromosome. *tetV* and *tet33* encode efflux proteins, known as membrane-associated proteins, that export tetracyclines from the cell. The export of tetracyclines reduces intracellular drug concentration and protects the ribosome within the cell. This may be a reason why strain OTC-16 grew well under tetracycline and OTC. As no research reported the *tet* genes were able to decompose tetracycline antibiotics, the role of these genes in strain OTC-16 during tetracycline degradation should be considered in the future. The gene *ant2ia* encodes aminoglycoside transferases, which inactivate aminoglycosides and confer resistance to aminoglycoside antibiotics^16^. In antimicrobial susceptibility testing, strain OTC-16 showed high resistance to streptomycin and medium resistance to tobramycin and kanamycin. *sul1* and *cml_e8* provided strain resistance to sulfonamides and chloramphenicol, respectively. The antibiotic resistance spectrum experiment in this work also confirmed the existence and function of these ARGs. Strain OTC-16 grew well under sulfadimethazine, sulfamethoxazole, sulfamonomethoxine, and cotrimoxazole and showed tolerance to chloramphenicol to some extent.

A total of three *A. nicotianae* strains have been sequenced to date. Strain ZM05 (accession number NZ_CP059853.1) has only one plasmid (accession number NZ_CP059854.1), and strain NBRC14234 (accession number NZ_BJNE00000000.1) has none. To compare the differences in the resistance genes carried by the three strains, the ARGs listed in Table S1 were used as target genes to BLAST among the three strains with the National Center for Biotechnology Information (NCBI) software. BLAST results showed that the ARGs contained in the three strains differed. Although the ARGs were mostly located in plasmids in strain OTC-16, they all existed on chromosomes in strains ZM05 and NBRC14234. Strain ZM05, isolated from soil, shared seven single-copy homologous resistance genes with strain OTC-16. The gene with the highest homology was *vanre*, with an identity value of 100%, followed by *vanrc* (99.41%) and *cara* (99.24%). While strain NBRC14234, isolated from sludge, had fewer ARGs, only the single-copy genes *bcra* and *ctab*3 were detected, and the homology was lower than that of strain ZM05 (Table S2). Other detailed information can be found in Supplementary Information. Comparison of the sequences of the three strains confirmed that the evolution of ARGs of the same bacterial species in different regions was different. Common ARGs are more likely to evolve in similar and geographically close surroundings, with little relativity to matrices where host bacteria survive. In this study, strains OTC-16 and ZM05 were both from China; they shared seven common ARGs. Meanwhile, strain NBRC14234, isolated from Japan, had only two low homologous common ARGs with strain OTC-16, although strains NBRC14234 and OTC-16 were both isolated from sludge. Therefore, ARGs carried by strains reflected the evolution of the strain in the environment and possibly reflected the antibiotic pollution levels under which the strains survived.

Plasmids with ARGs replicated independently in cells and could be transferred to other bacteria through transformation and conjugation, leading to gene transport and diffusion. In contrast, in strains with ARGs distributed on chromosomes, such as strains ZM05 and NBRC14234, the transmission of ARGs may be mainly achieved by transposition.

### Expression of ARGs in OTC-16 under OTC stress

**Figure 3:**
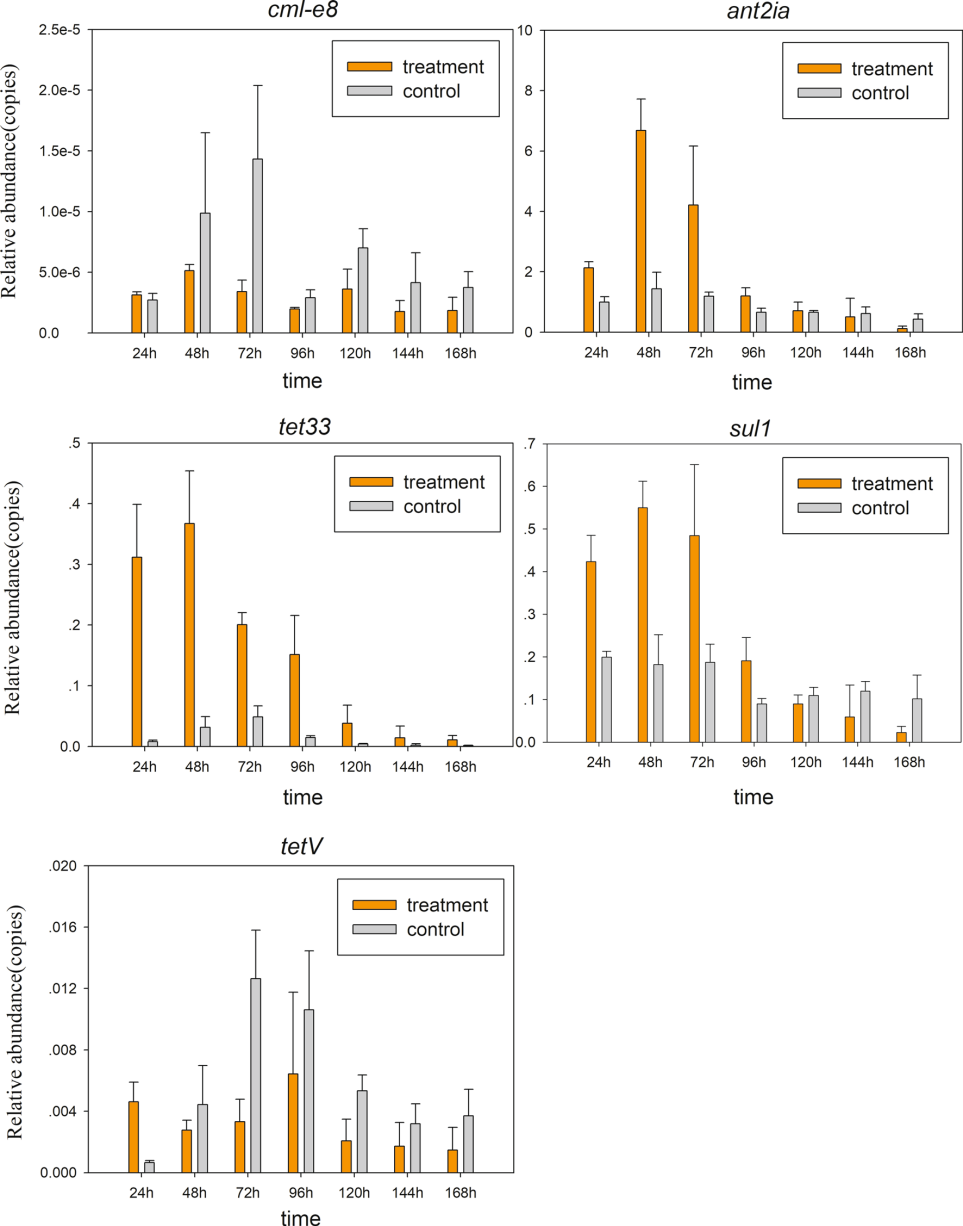
Relative abundance of resistance gene expression at 24–168 h.

**Figure 4:**
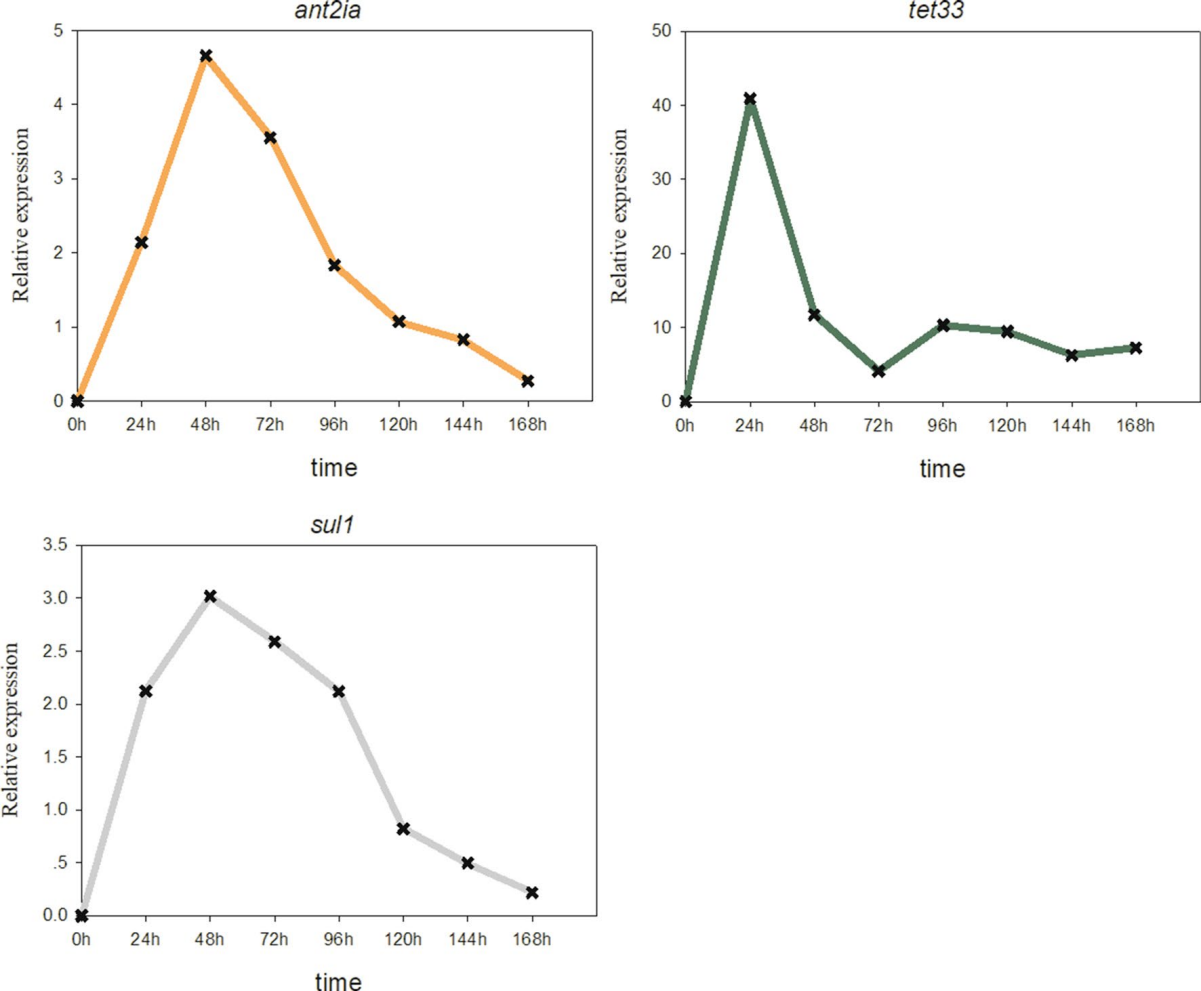
The expression levels of resistance genes *tet33*, *sul1*, and *ant2ia* changed at 24–168 h.

**Figure 5:**
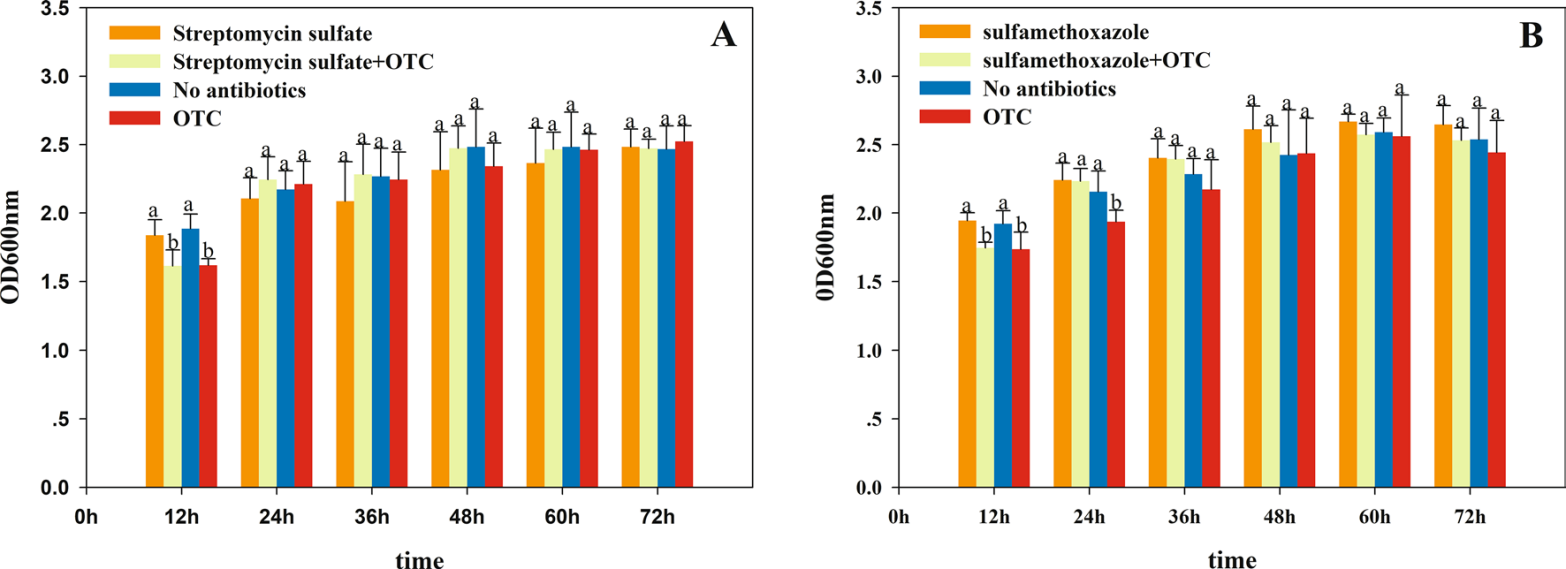
Effect of streptomycin sulphate (**A**) and sulfamethoxazole (**B**) on the growth of strain OTC-16. Different letters above error bars indicate significant differences (*P* < 0.01) among treatments.

To study the expression characteristics of ARGs at different genetic loci, the behaviour of *sul1*, *tet33*, *ant2ia*, and *cml_e8*, located in plasmids and *tetV* carried by chromosomes, were identified using qRT-PCR. The results showed that all five resistant genes could be expressed in strain OTC-16 with or without exposure to OTC. The expression levels of these genes increased at first and then decreased during incubation; however, the peak time was slightly different (Fig. 3). Thus, the five tested ARGs were identified as constitutively expressed genes, which were automatically expressed with the growth of bacteria regardless of resistant stress. Nonetheless, the expression levels of these genes were significantly different. *ant2ia* expression was dominant, and its relative expression value was approximately 10 times that of *tet33* and *sul1*, 1,000 times that of *tetV*, and 10^5^ times that of *cml_e8*. Overall, *ant2ia*, *tet33*, and *sul1* were more active in strain OTC-16 than *tetV* and *cml_e8.* The antibiotic resistance spectrum experiment in this study confirmed the existence and expression of these ARGs. Strain OTC-16 grew well in the presence of streptomycin, tetracycline, oxytetracycline, sulfadimethazine, and sulfamethoxazole. Nevertheless, the appearance of chloramphenicol inhibited the cell proliferation of this strain, possibly because of the low expression of gene *cml_e8*. Figure 4 indicates that the presence of OTC generally suppressed the expression of *cml_e8* and *tetV* but significantly promoted the relative expression of *ant2ia*, *sul1*, and *tet33* during early culture (0–48 h). The maximum up-regulated expression occurred at 24 h for *tet33* and 48 h for *ant2ia* and *sul1*; however, they all declined sharply 72 h later. The expression of *ant2ia* and *sul1* at 168 h in OTC treatments was even lower than that in the controls. Overall, OTC evidently up-regulated the expression of these ARGs transiently, and the short-term up-regulation effect might play an important role in the biodegradation of OTC by strain OTC-16. Our previous studies confirmed that the presence of OTC inhibited early growth and reduced the biodegradation rate of strain OTC-16 at the initial stage^12^, but biodegradation improved rapidly 48 h later with a high expression of ARGs. The up-regulation of genes *sul1* and *ant2ia* by OTC (Fig. 4) implied that the presence of OTC improved the resistance of strain OTC-16 to antibiotics encoded by these genes. The hypothesis was tested using the experiment shown in Fig. 5, in which the growth of *A. nicotianae* OTC-16 in different antibiotic treatments is shown. It was found that OTC is more toxic to strain OTC-16 than streptomycin sulphate and sulfamethoxazole. The cell growth of strain OTC-16 in the presence of streptomycin sulphate and sulfamethoxazole was markedly better than that in the presence of OTC. Although the co-existence of streptomycin sulphate and OTC resulted in decreased cell growth of strain OTC-16 in the early stage, the adverse effect changed after 24 h, which is likely related to the up-regulation of *ant2ia* (Fig. 4). In addition, for extended periods, the cell number of strain OTC-16 in the streptomycin sulphate–OTC system was higher than that in the presence of streptomycin sulphate alone, indicating that the presence of OTC improved the resistance of the strain to streptomycin sulphate. In contrast, the co-existence of sulfamethoxazole and OTC always resulted in lower cell growth of strain OTC-16 than sulfamethoxazole alone, although the adverse effect was alleviated after 24 h. Using the tetracycline-resistant bacterium *Shigella flexneri* as a research subject, Gao^19^ found that tetracycline stress inhibited the strain and the growth rate of the bacterial concentration decreased with increasing tetracycline concentration. Moreover, tetracycline stress promoted the expression of some tetracycline resistance genes in *S. flexneri*. However, Gao claimed no significant correlation between tetracycline concentration and abundance of tetracycline-resistance genes and their expression levels. Our study results are consistent with Gao’s conclusion that antibiotics inhibit growth and promote ARG expression in antibiotic-resistant strains. The correlation between antibiotic concentration and ARG expression levels need further study. In this study, the presence of OTC could also activate other ARGs of strain OTC-16 not related to OTC resistance, such as the sulphonamide resistance gene *sul1* and aminoglycoside resistance gene *ant2ia.* However, the resistance of the strain to these antibiotics is not absolutely improved and might depend on many factors, such as the type of antibiotics and duration.

**Figure 6:**
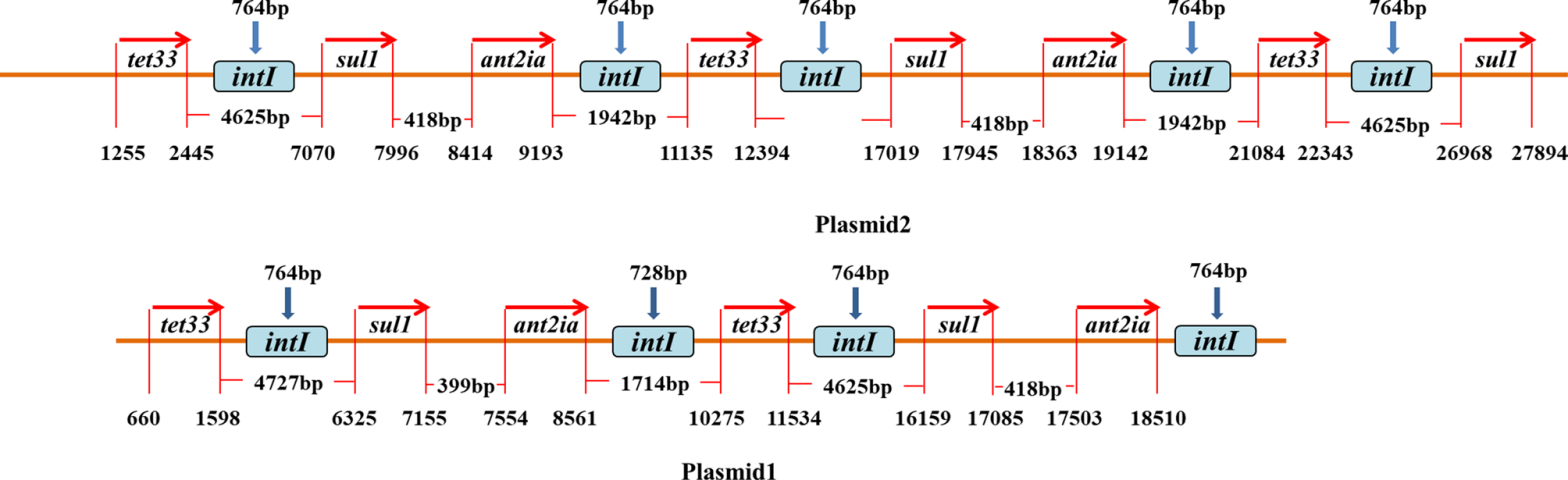
The sites of gene *sul1*, *tet33* and *ant2ia* on plasmid 1 and plasmid 2.

The synchronised up-regulated expression of *tet33*, *sul1*, and *ant2ia* indicates that the three ARGs may have a synergistic regulation mechanism. Synergistic regulation refers to the process in which a series of transcription and translation systems of bacteria respond simultaneously to any kind of environmental pressure, such as antibiotics and heavy metals^20^. Through this mechanism, the addition of OTC can reduce the sensitivity of strain OTC-16 to other antibiotics. A similar phenomenon has been reported for heavy metals; for example, copper and zinc ions can coordinate the regulation of ARGs and enhance bacterial tolerance to chloramphenicol, tetracycline, thiomycin, and other antibiotics^21–23^. In addition, similar fluctuation trends and the synchronous transcription phenomenon among the three ARGs suggest that they may have relatively close genetic loci or may be regulated by the same or similar regulatory proteins. Analysing and mapping the gene drafts of strain OTC-16 showed that *ant2ia, sul1*, and *tet*33 were all multiple copy genes, and these genes were closely connected in position and formed tandem repeat *tet*33-*sul1-ant2ia* gene series located exclusively in plasmids 1 and 2. Moreover, constant spacer regions of 4625 bp were observed between *tet*33 and *sul1*, 418 bp were observed between *sul1* and *ant2ia*, and 1492 bp were observed between *ant2ia* and *tet*33 (Fig. 6). Based on the expression pattern and gene location, we speculated that these three genes may be in a resistance chain transmission vector, such as transposon and integron, and could be transferred horizontally^24^. Integrons are genetic entities that are defined by their ability to capture members of a large family of small mobile elements known as ‘gene cassettes’. Gene cassettes can be incorporated into a specific site of integrons by a site-specific recombinase encoded by the gene *intI*^25^. To verify our speculation, the sequence of *intI* was examined in the plasmids of strain OTC-16. Finally, a total of 10 copies of *intI*, mostly with a constant sequence region (764 bp), were recognised. All genes were located adjacent to the ARGs in the cassettes mentioned above; four were located in plasmid 1 and six in plasmid 2 (Fig. 6). Integrons are a common genetic element involved in the development of multiple antibiotic resistance and play vital roles in improving the potential of bacteria to adapt to the surrounding environments^26,27^. The survival of bacteria sometimes depends on their ability to capture integrons and transferability of the same plasmid. In this respect, integrons are products of the evolution of bacteria.

Plasmid-derived ARGs (*ant2ia*, *sul1*, and *tet*33) were greatly induced by external addition of OTC and had obviously higher expression activity than genes carried by chromosomes (*tetV*). The results are consistent with the report by Qin^28^, who claimed that genes associated with mobile genetic elements generally showed higher expression levels under antibiotic-treated conditions than genes in chromosomes, based on transcriptomics analysis of 12 multidrug-resistant *Acinetobacter baumannii*. The hypothesis was further verified by Liu^29^, who conducted metagenome and macro-transcriptome analyses on sludge and found that ARGs with high transcription abundance are mainly related to plasmid-derived resistance genes. We hypothesise that the tetracycline-specific efflux pump gene *tet33* of the major facilitator superfamily on the plasmids may be an important genetic element for strain OTC-16 to resist OTC; however, its role in the degradation process of OTC needs to be further verified. The inherent ARGs improve the resistance of the degrading strain to various antibiotics and enhance its adaptability to the environment when the host bacterium was applied as a bioaugmentation/biodegradation agent. Nonetheless, the presence of these genes also increases the risk of antimicrobial resistance expansion, especially those located in mobile genetic elements such as plasmids and integrons, similar to *ant2ia, sul1*, and *tet*33 in this study. Plasmid conjugation is one of the dominant horizontal gene transfer mechanisms^30,31^ contributing to the increasing resistance of indigenous bacteria. A recent study showed that a conjugative antibiotic resistance plasmid, RP4, harboured by the donor strain *Pseudomonas putida* KT2442 could be disseminated to bacteria affiliated with more than 15 phyla^32^. Therefore, from this point of view, strain OTC-16 may currently pose a high ecological threat, mainly owing to the resistance plasmids. Nevertheless, despite the ecological risks, one cannot deny the significant application prospects of the strain. The expression behaviour of the resistant genes in this study provides some insights into the genetic modification of the strain. As most resistance genes are located on plasmids, a strategy capable of bypassing the development of resistance by shearing redundant resistance genes on plasmids and even directly eliminating plasmids can be adopted in the future to reduce the proliferation and spread of resistance genes in the environment. Some recent researches on the bacteriostasis of resistant strains and plasmids curing have been performed. The antimicrobial and anti-biofilm effects of bioactive compounds from *Psammocinia* sp. and *Hyattella* sp. sponges toward iatrogenic, antibiotic-resistant bacteria have been reported^33^. Bioactive metabolites extracted from sea anemone (*Stichodactyla haddoni*), with antibacterial and anti-biofilm activities against antibiotic-resistant bacteria, were also screened and identified^34^. Besides, Hassanshahian^35^ reported a successful case of plasmid curing by developing nanoemulsion production from the essential oil of *Alhagi maurorum*. The nanoemulsion had a remarkable effect on the curing of resistant plasmids of three antibiotic-resistant bacteria. The success of these studies provides us with an alternative strategy for the modification and safe use of the OTC-degrading strain OTC-16.

Antibiotic-degrading strains are good bioaugmentation/bioremediation agents in remediating antibiotic-contaminated environments. The benefits of using antibiotic-degrading bacteria to manage antibiotic pollution should be assessed against the risk of the potential expansion of antimicrobial resistance. Nevertheless, investigation on antibiotic resistance pattern and intrinsic mechanism of the potential antibiotic-degrading strains are rarely reported. This study explicated the antibiotic resistance behaviour of the antibiotic-degrading strain OTC-16 from a genomics perspective. The results will deepen our understanding of the resistance pattern of antibiotic-degrading strains and elucidate the role of ARGs in antibiotic resistance and adaptive evolution.

## Conclusions

Many ARGs unrelated to tetracycline degradation were identified in an efficient OTC/tetracycline-degrading bacterium *A. nicotianae* OTC-16, making the strain resistant to at least seven categories of 15 antibiotics. Actively expressed genes, such as *ant2ia*, *sul1,* and *tet33*, are mostly located in plasmids and are incorporated into integrons as ‘gene cassettes’ with obvious mobile characteristics. This study provides valuable information for the improved and safe application of this strain as well as insights into reducing its ecological risk via genetic modification. These findings deepen our understanding of the ecological role and adaptive evolution of antibiotic-degrading microorganisms in the environment.

## Materials and methods

### Strain and medium

The OTC-degrading strain *A. nicotianae* OTC-16, used in this study, was previously isolated from active sludge around a pharmaceutical factory in Taizhou, Zhejiang province, China. Luria–Bertani (LB) medium (pH 7.2) containing 10 g L^−1^ tryptone, 5 g L^−1^ yeast extract, and 10 g L^−1^NaCl was used to incubate the strain. The kits used in this study included the AxyPrep Plasmid Miniprep Kit, RNAiso Plus Kit (Takara Biochemicals, Hangzhou, China), DNA Extraction Kit (TransGen Biotech, Beijing, China), PrimeScript RT Reagent Kit with gDNA Eraser (Takara Biochemicals, Hangzhou, China), and MightyAmp for Real Time Kit (TB Green Plus; Takara Biochemicals, Hangzhou, China). The manufacture’s instructions were followed.

### Antimicrobial susceptibility testing

The susceptibility of the OTC-16 strain to drugs was determined using the disk diffusion method^33^ with slight modifications. A single colony of strain OTC-16 was inoculated into LB liquid medium, and 80 μL of bacterial suspension grown to an optical density (OD) of 1.6 at 600 nm (10^8^ CFU mL^−1^) was spread evenly on the LB solid medium in Petri dishes. Paper disks soaked with antibiotics were then placed in these plates. The inhibitory effects of antibiotics on strain OTC-16 were assessed after 24 h by measuring the diameter of the bacteriostatic circle of each disk. The absence of a bacteriostatic circle indicated that the strain was highly resistant to the target drug; the appearance of an obvious bacteriostatic circle (diameter ≥ 1.3 cm) indicated that the strain was sensitive to the antibiotic. When the diameter of the bacteriostatic circle was less than 1.3 cm, the strain was deemed to have medium resistance to the drug. The experiment was repeated three times and performed in triplicates for each drug, with a total of 50 commonly used antibiotics tested. The antibiotics-soaked paper disks tested in this experiment (diameter: 6 mm) were purchased from Solarbio (Beijing, China). The concentration unit for the tested drugs was generally μg per disk unless indicated. The detailed information about drugs and concentrations tested is presented in Table 1.

### Whole-genome sequencing

*A. nicotianae* OTC-16 was cultivated aerobically at 30 °C for 24 h in LB medium. DNA extraction was conducted using the DNA extraction kit according to the manufacturer’s protocol. The genome of *A. nicotianae* OTC-16 was sequenced using a PacBio RS II platform and Illumina HiSeq 4000 platform at the Beijing Genomics Institute (BGI, Shenzhen, China). The tRNAs and rRNAs were identified using tRNAscan-SE^36^, RNAmmer^37^, and Rfam databases. The best hit was abstracted using the BLAST alignment tool for function annotation. Seven databases, including KEGG^38,39^, Cluster of Orthologous Groups of proteins (COG), Non-Redundant Protein Sequence Database (NR), Swiss-Prot, GO, Translation of European Molecular Biology Laboratory (TrEMBL), and Evolutionary Genealogy of Genes: Non-supervised Orthologous Groups (EggNOG) were used for general function annotation. Virulence factors and resistance genes were identified based on the core dataset in the Virulence Factors of Pathogenic Bacteria (VFDB) and the CARD.

### Quantitative reverse transcription-polymerase chain reaction

To elucidate the role of the ARGs of strain OTC-16 in antibiotic resistance under OTC, five ARGs (*tetV*, *sul1*, *tet33*, *ant2ia*, and *cml_e8*) distributed in chromosome and plasmids were randomly selected with reference to the whole genome sequence of *A. nicotianae* strain OTC-16 (NZ_CP 033 081), and the expression of these ARGs was detected using real-time quantitative reverse transcription-polymerase chain reaction (qRT-PCR). Freshly cultured bacterial suspensions with OD_600nm_ = 1.8 were inoculated into treatment (LB broth spiked with 100 mg L^−1^ OTC, hereafter referred to as LB^R^ broth) and control (LB broth only) at a dose of 3% (v/v) and then incubated at 30 °C for 7 d. The cultures were performed in triplicates, and the bacterial suspensions were sampled every 24 h. Total RNA was extracted using the RNAiso plus Kit. The obtained RNA was dissolved in ribonuclease-free water, and high-quality RNA with purity and concentration, which met the PCR standard, was saved and used in the subsequent experiments. Approximately 1 μg of RNA was used for real-time PCR in each sample. Complementary DNA (cDNA) was synthesised using a PrimeScript RT reagent kit, according to the manufacturer’s instructions. The primers for the candidate genes were designed using the primer analysis software Primer 5.0, and the primer specific verification was performed on the NCBI database. The 16S rRNA V3 region (simply referred to as 16S rRNA) was used in the study as the reference gene for quantitative analysis. Detailed information on the primers is listed in Table 2.

**Table 2:** Primers and annealing temperatures for qPCR assays.

Primer	Primer sequence (5′–3′)	T_m_ (℃)	Annealing time
*tet33-F*	TCCCGCACTGCTACACGA	55	15 s
*tet33-R*	GCGAGATGGCACCGAACA		
*cml_e8-F*	CGTGCCAGCGAACCAGAAG	55	15 s
*cml_e8-R*	TGGGTGCCAGGAAGGTGAAT		
*tetV-F*	GCACTCGTCTTCTCCACCTT	55	15 s
*tetV-R*	GGTTCCCTCCAGCCCATTAG		
*ant2ia-F*	CATCCCGTGGCGTTATCC	55	15 s
*ant2ia-R*	CTGGGCAGGTAGGCGTTT		
*sul1-F*	CGCACCGGAAACATCGCTGCAC	65	15 s
*sul1-R*	TGAAGTTCCGCCGCAAGGCTCG		
*16S rRNA-F*	CCTACGGGAGGCAGCAG	62	15 s
*16S rRNA-R*	ATTACCGCGGCTGCTGG		

Real-time qRT-PCR was performed with Mighty Amp for Realtime PCR Kit and conducted on an ABI StepOnePlus Real-Time PCR System (PerkinElmer Applied Biosystems, Foster City, CA, USA). The specificity of the amplified product was confirmed by the melting temperature. With 16S rRNA as the internal reference gene, the results of the qRT-PCR were normalised to obtain the relative value of resistance gene expression. The relative value of gene expression and the relative expression of resistance genes were calculated using the following two formulas:

1 $${\text{Relative value of gene expression}} = \frac{N1}{{N2}}$$

2 $${\text{Relative expression of resistance gene}} = \frac{VT}{{VCK}}$$

where *N1* denotes the copy number of the target gene, *N2* denotes the copy number of the 16S rRNA gene, *VT* denotes the gene expression value of treatment group genes, and *VCT* denotes the gene expression value of the control group genes.

### Response of cell growth to co-existence of multiple antibiotics

Freshly cultured bacterial suspensions with OD_600nm_ = 1.8 were inoculated into 5 mL of LB^R^ broth at a 3% (v/v) dose. Streptomycin sulphate and sulfamethoxazole were selected as target antibiotics in this experiment at a final concentration of 100 mg L^−1^. Four groups were designed for each target antibiotic (TA): (1) TA + strain OTC-16; (2) TA + OTC + strain OTC-16; (3) OTC + strain OTC-16; and (4) strain OTC-16 only. The cultures were performed in triplicates, and the OD_600nm_ values of the bacterial suspension were recorded every 12 h.

Differences in cell growth (OD_600nm_) among treatments were tested with Duncan's test using analysis of variance on SPSS version 16.0 for Windows. Differences were considered significant if the P value was less than 0.01.

## Supplementary Information

Supplementary Information.
