# Supplementary material for: Genomic insights into the antibiotic resistance pattern of the tetracycline-degrading bacterium, Arthrobacter nicotianae OTC-16
Source: Sci Rep. 2021 Aug 2;11:15638. doi: 10.1038/s41598-021-94840-y (PMC8329189; doi:10.1038/s41598-021-94840-y)
Supplement: Supplementary file 1 — Supplementary Information. [file 41598_2021_94840_MOESM1_ESM.docx]

**Genomic insights into the antibiotic resistance pattern of the tetracycline-degrading bacterium, *Arthrobacter nicotianae* OTC-16**

Xin Zhang^a,#,*^, Rongrong Zhu^a,b,#^, Weilin Li^a,b^, Junwei Ma^b^ , Hui Lin^b,*^,

^a^ *College of Forest and Biotechnology, Zhejiang A & F University, Hangzhou 311300, China*

*^b^ The Institute of Environment, Resources, Soil and Fertilizers, Zhejiang Academy of Agricultural Sciences, Hangzhou 310021, China*

*^#^ Equal contributions thus sharing first authorship.*

**^*^ Corresponding authors at:** College of Forest and Biotechnology, Zhejiang A&F University, Tel: +86-571-61069780, E-mail address: zhangxins@126.com (X. Zhang); The Institute of Environment, Resources, Soil and Fertilizers, Zhejiang Academy of Agricultural Sciences, Tel: +86-571-86404302, Fax: +86-571-86404302, E-mail address: linhui@zaas.ac.cn (H. Lin)

**Captions**

Table S1 ARGs information of strain OTC-16

Table S2. The distribution of ARGs among sequenced *Arthrobacter nicotianae* strains

Figure S1 GO function annotation distribution map of strain OTC-16

**Table S1** ARGs information of strain OTC-16

| Location | Gene_id | Subject_id | Gene_  type | Antibiotic_  resistance | Original_  Resistance_Type |
| --- | --- | --- | --- | --- | --- |
| Chromosome | ZAF-05GL000241 | ardb_2199 | *tetv* | tetracycline | tet_efflux |
| Chromosome | ZAF-05GL000615 | ardb_115 | *vanre* | vancomycin | vane |
| Chromosome | ZAF-05GL000913 | ardb_1951 | *cml_e6* | chloramphenicol | cml |
| Chromosome | ZAF-05GL001175 | ardb_2604 | *cara* | lincosamide,streptogrammacrolide | mls_abc |
| Chromosome | ZAF-05GL001392 | ardb_2322 | *bcra* | bacitracin | bcr |
| Chromosome | ZAF-05GL001549 | ardb_2778 | *catb3* | chloramphenicol | catb |
| Chromosome | ZAF-05GL002124 | ardb_2395 | *vanrc* | vancomycin | vanc |
| Chromosome | ZAF-05GL003407 | ardb_2606 | *bcra* | bacitracin | bcr |
| Plasmid1 | ZAF-05GL003435 | ardb_598 | *tet33* | tetracycline | tet_efflux |
| Plasmid1 | ZAF-05GL003441 | ardb_571 | *sul1* | sulfonamide | sul |
| Plasmid1 | ZAF-05GL003442 | ardb_2203 | *ant2ia* | tobramycin,kanamycin,  sisomicin,dibekacin,  gentamicin | ant |
| Plasmid1 | ZAF-05GL003445 | ardb_598 | *tet33* | tetracycline | tet_efflux |
| Plasmid1 | ZAF-05GL003450 | ardb_571 | *sul1* | sulfonamide | sul |
| Plasmid1 | ZAF-05GL003451 | ardb_2203 | *ant2ia* | tobramycin,kanamycin,  sisomicin,dibekacin,  gentamicin | ant |
| Plasmid1 | ZAF-05GL003527 | ardb_1888 | *cml_e8* | chloramphenicol | cml |
| Plasmid2 | ZAF-05GL003540 | ardb_598 | *tet33* | tetracycline | tet_efflux |
| Plasmid2 | ZAF-05GL003544 | ardb_571 | *sul1* | sulfonamide | sul |
| Plasmid2 | ZAF-05GL003545 | ardb_2200 | *ant2ia* | tobramycin,kanamycin,  sisomicin,dibekacin,  gentamicin | ant |
| Plasmid2 | ZAF-05GL003548 | ardb_598 | *tet33* | tetracycline | tet_efflux |
| Plasmid2 | ZAF-05GL003552 | ardb_571 | *sul1* | sulfonamide | sul |
| Plasmid2 | ZAF-05GL003553 | ardb_2200 | *ant2ia* | tobramycin,kanamycin,  sisomicin,dibekacin,  gentamicin | ant |
| Plasmid2 | ZAF-05GL003556 | ardb_598 | *tet33* | tetracycline | tet_efflux |
| Plasmid2 | ZAF-05GL003560 | ardb_571 | *sul1* | sulfonamide | sul |

**Table S2** The distribution of ARGs among sequenced *Arthrobacter nicotianae* strains

| Gene_id | Gene_  type | Gene_Location (gene identity) | | | | |
| --- | --- | --- | --- | --- | --- | --- |
|  |  | Strain OTC-16 | | Strain ZM05 | | StrainNBRC14234 |
|  |  | Chromosome | Plasmid | Chromosome  （Identities %） | Plasmid | Chromosome  （Identities %） |
| ZAF-05GL000241 | *tv* | + |  | + (96.78%) |  |  |
| ZAF-05GL000615 | *vanre* | + |  | + (100% ) |  |  |
| ZAF-05GL000913 | *cml_e6* | + |  | + ( 98.56%) |  |  |
| ZAF-05GL001175 | *cara* | + |  | + (99.24% ) |  |  |
| ZAF-05GL001392 | *bcra* | + |  | + (94.25% ) |  | + (94.17% ) |
| ZAF-05GL001549 | *catb3* | + |  | + (98.44% ) |  | + (98.23% ) |
| ZAF-05GL002124 | *vanrc* | + |  | + (99.41% ) |  |  |
| ZAF-05GL003407 | *bcra* | + |  |  |  |  |
| ZAF-05GL003435 | *tet33* |  | + |  |  |  |
| ZAF-05GL003441 | *sul1* |  | + |  |  |  |
| ZAF-05GL003442 | *ant2ia* |  | + |  |  |  |
| ZAF-05GL003445 | *tet33* |  | + |  |  |  |
| ZAF-05GL003450 | *sul1* |  | + |  |  |  |
| ZAF-05GL003451 | *ant2ia* |  | + |  |  |  |
| ZAF-05GL003527 | *cml_e8* |  | + |  |  |  |
| ZAF-05GL003540 | *tet33* |  | + |  |  |  |
| ZAF-05GL003544 | *sul1* |  | + |  |  |  |
| ZAF-05GL003545 | *ant2ia* |  | + |  |  |  |
| ZAF-05GL003548 | *tet33* |  | + |  |  |  |
| ZAF-05GL003552 | *sul1* |  | + |  |  |  |
| ZAF-05GL003553 | *ant2ia* |  | + |  |  |  |
| ZAF-05GL003556 | *tet33* |  | + |  |  |  |
| ZAF-05GL003560 | *sul1* |  | + |  |  |  |

+：The ARGs exist in the location; Identities %: the homology of ARGs of other sequenced *Arthrobacter nicotianae* strains compared with the strain OTC-16.


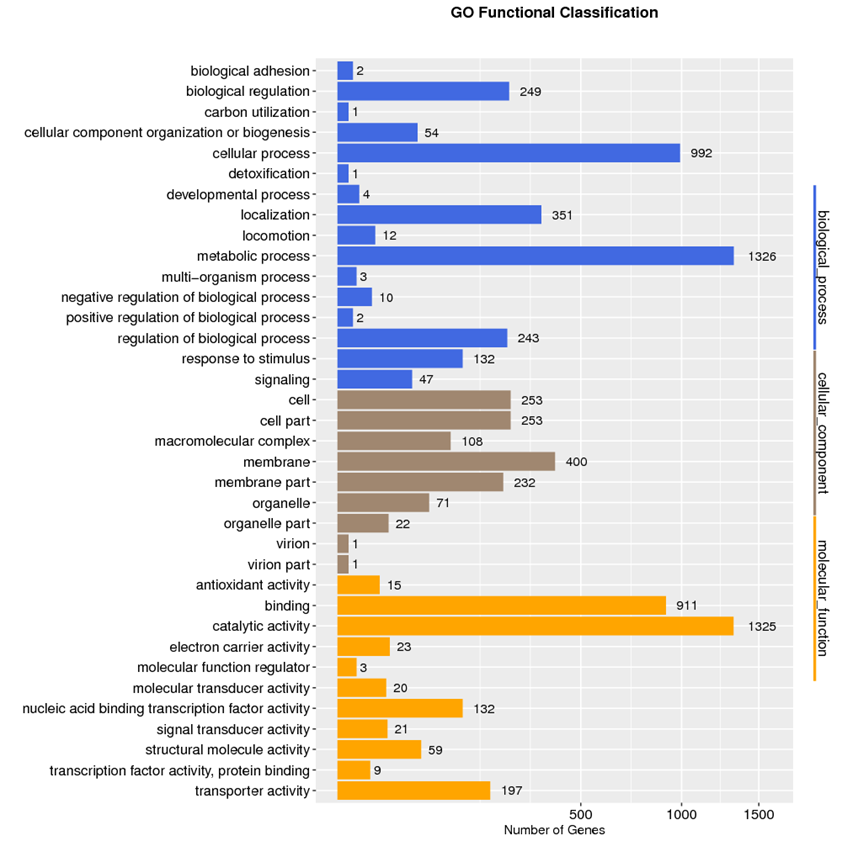


**Figure S1** GO function annotation distribution map of strain OTC-16
